# Social Acceptance of Smart Glasses in Health Care: Model Evaluation Study of Anticipated Adoption and Social Interaction

**DOI:** 10.2196/49610

**Published:** 2025-02-11

**Authors:** Niek Zuidhof, Oscar Peters, Peter-Paul Verbeek, Somaya Ben Allouch

**Affiliations:** 1 Research Group Technology, Health & Care Saxion University of Applied Sciences Enschede The Netherlands; 2 Research Group Employability Transition Saxion University of Applied Sciences Enschede The Netherlands; 3 School of Finance and International Business Saxion University of Applied Sciences Enschede The Netherlands; 4 Philosophy and Ethics of Science and Technology in a Changing World University of Amsterdam Amsterdam The Netherlands; 5 Research Group Digital Interactions University of Amsterdam Amsterdam The Netherlands; 6 Research Group Digital Life Amsterdam University of Applied Sciences Amsterdam The Netherlands

**Keywords:** smart glasses, technology adoption, social interaction, instrument development, structural equation modeling

## Abstract

**Background:**

Despite the growing interest in smart glasses, it is striking that they are not widespread among health care professionals. Previous research has identified issues related to social interactions involving the use of smart glasses in public settings, which may differ from those associated with their application in health care contexts.

**Objective:**

Assuming that smart glasses mediate contact between the health care provider and patient, the objectives of this research are two-fold: (1) to develop an instrument that combines the adoption and mediation perspectives, and (2) to gain insights into how the intention to use is influenced through aspects of adoption and social interaction.

**Methods:**

A questionnaire was administered to a target audience of health care professionals (N=450), with recruitment via MTurk. The sample primarily included male participants from the United States, with the majority aged 42 years or younger. Although a large portion of respondents were medical doctors, the sample also included nurses and other health care professionals. Data were analyzed by structural equation modeling.

**Results:**

Regarding the aim of developing an instrument combining adoption and social interaction, the internal consistency was above the aspirational level (α>.70) for the instrument. Furthermore, regarding the second objective involving gaining insights into the influential constructs of the anticipated intention to use, the following results were highlighted: in testing the conceptual model, the measurement model generated a good fit and the respecified structural model also generated a good fit. The tested hypotheses confirmed that social interaction constructs could explain a higher variance of users’ anticipated intention to use. Perceived social isolation and decreased attentional allocation did not have a significant effect on attitude. Furthermore, the intention to use smart glasses despite nonacceptance of smart glasses by the patient significantly influenced the anticipated intention to use. In summary, constructs that focus on social interaction could contribute to better explanation and prediction of the expected adoption of smart glasses in health care.

**Conclusions:**

The empirical findings of this study provide new insights into how the mediation perspective can increase the explained variance compared to existing knowledge about adoption. Against expectations based on previous literature and despite the social issues raised earlier, these social aspects do play important roles for health care professionals but are ultimately not decisive for the intention to use. As a result, there are fewer threats to the adoption of smart glasses from the perspective of health care professionals than might be expected based on the previous literature. Therefore, the use of smart glasses can still be considered as an innovative way of working in health care.

## Introduction

### Background

**Figure 1:**
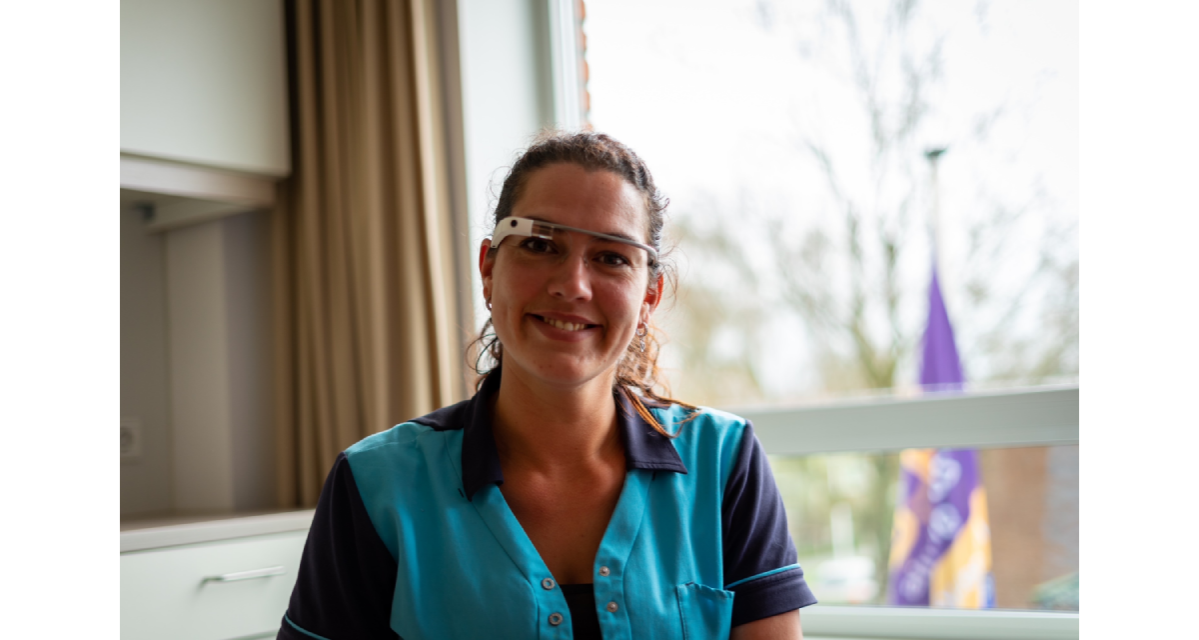
Example of a health care professional wearing Google Glass smart glasses in a home environment to illustrate the appearance of the glasses.

The initial public reactions to smart glasses, notably Google Glass (Figure 1), were quite mixed. Google Glass was both praised for its innovative technologies, such as a see-through screen and hands-free use, and criticized for various reasons. Shortly after the introduction of Google Glass, cautionary stories about “Glassholes” emerged, a term coined to describe individuals who misuse the technology in socially inappropriate ways [1]. Examples of this phenomenon include people who wear Google Glass and ignore the people around them or stare off into space while operating their smart glasses. Google responded with rules to help users avoid these situations [2]. There are several examples of various emotional reactions to smart glasses in the media, and ethical questions are repeatedly raised about wearing smart glasses [3,4]. Although research into smart glasses is continuously expanding, current knowledge and theoretical foundations about the acceptance of smart glasses are still scarce.

Smart glasses have emerged as a growing research topic over the past years in various fields [5,6] and could bring about a revolution in health care [7]. Smart glasses are computerized head-mounted devices worn like eyeglasses, which can collect and provide information to the user and the environment. The majority of smart glasses incorporate a display through which information can be accessed while simultaneously maintaining visibility of the external environment. Furthermore, these devices often provide the advantage of hands-free operation [8]. Pilot studies on the use of smart glasses in health care have been conducted [7]; however, few researchers have addressed the issue of preadoption criteria, and identifying these criteria remains a critical issue [9].

### Prior Research on the Adoption of Smart Glasses

The initial responses to smart glasses raise the question of how the adoption of smart glasses is progressing.

The growing body of research on the adoption of smart glasses was searched in the existing scientific literature. The following keywords were used: “smart glasses,” “head-mounted display*,” “wearable display*,” “augmented reality (smart) glasses,” “adoption,” “acceptance,” and “acceptability.” The search was performed in the following databases on June 12, 2021: Scopus (11 hits), Web of Science (12 hits), ACM Digital Library (96 hits), IEEE Xplore (2 hits), and PubMed (0 hits). All synonyms were connected with the disjunction “OR,” and both “smart glasses” and “adoption” were connected with the conjunction “AND.” Keywords were searched in the title. The identified publications are listed in Multimedia Appendix 1.

Various methodologies have been employed by researchers to investigate the adoption of smart glasses. For an overview of these methods, please refer to Multimedia Appendix 1. The context of the studies varied, with the general context most often consisting of random individuals or students. A small number of studies were found in the context of health care and tourism; therefore, it can be concluded that knowledge about the adoption of smart glasses is still scarce in these fields. Adoption studies with industry, retail, and agriculture respondents are even more limited. Various methods have been applied, with the majority of studies being qualitative in nature. Most of the previous quantitative studies had a relatively low number of respondents. The findings presented in Multimedia Appendix 1 offer foundational inputs for the development of a questionnaire. Furthermore, the social aspects surrounding smart glasses were mentioned multiple times in previous studies, and therefore, it could be assumed that social interaction is a relevant topic regarding the acceptance of smart glasses.

The Unified Theory of Acceptance and Use of Technology (UTAUT) [10], the Technology Acceptance Model (TAM) [11], and the Diffusion of Innovations [12] were found to be the most commonly accepted theories by scholars [13,14]. Some of the findings from prior research in Multimedia Appendix 1 might be related to the aforementioned models such as the influence on perceived usefulness, perceived ease of use, facilitating conditions, image, and perceived enjoyment [10,15]. However, other findings suggest themes regarding both the ethical and social contexts, which can be termed social acceptability [16]. Social acceptability can be divided into conversation partners, such as interpersonal communication, and spectators or third parties, with examples such as disturbance and controversial public use. The theme of privacy is also related to the social context and can be divided into the same categories: conversation partners and third parties. For example, wearing smart glasses is not only about the privacy of the user, but also about other people’s privacy, and this can strongly influence the user’s decision-making [17]. Figure 1 provides an illustrative example of an encounter with a health care professional wearing smart glasses. To summarize the findings of the prior research in Multimedia Appendix 1, it can be concluded that relevant aspects of the preadoption criteria should be informed by the current knowledge in adoption research, such as the TAM, and should also take into account the social context from the perspectives of both caregivers and patients.

Devices worn on the body in general influence the symbolic meaning in the natural environment of the person and the public surroundings and have psychological impacts on wearers and behavioral consequences for wearers [18]. Over the past decades, the TAM [19] had an incredible influence on empirical research. One might also say in general that current adoption models take a snapshot of adoption readiness, and the TAM consistently explains about 40% of the variance in the intention to use IT and the actual usage by individuals [15]. However, these adoption models have limitations [20], in which the current reality regarding smart glasses may not be fully involved. Current models are linear and strongly focused on the user’s perspective alone. Moreover, according to recent studies, social influence, as it is considered in the UTAUT [10], may involve more than just the influence and desire of others in the future use of the one considering it [3]. Several studies have suggested that social context influences usage norms and that public use is controversial [21,22]. The use of smart glasses does not reproduce existing norms and understandings in turn-taking, knowledge, and identity and could reconfigure norms by creating new settings in which activities begin to compete and interfere with each other [23,24]. In addition, privacy is often mentioned to be influential [25]. The privacy of the user is important, and other people’s privacy can also strongly influence the user’s decision-making [17]. Therefore, smart glasses force us to take a broader view of adoption models, and we should not only consider the perspectives of potential users but also look at the surroundings. Hence, social influences might be currently underestimated in the context of the acceptance of smart glasses given the reports from various media and recent publications on the subject.

**Figure 2:**
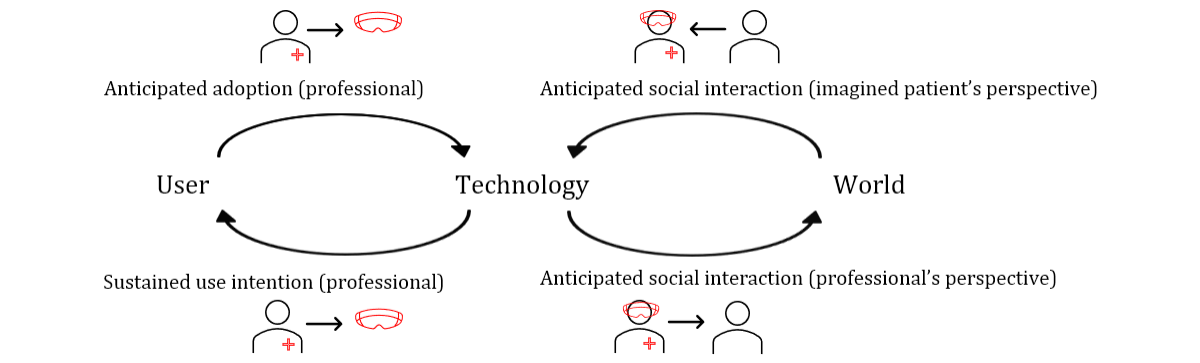
Theoretical framework of anticipated adoption and social interaction.

Turning to a perspective that takes a broader view of users and their surroundings and environment [26], the mediation perspective fits well with the identified gaps regarding the acceptance of smart glasses. The mediation perspective of the philosophy of technology considers the relationship between humans and technology from a phenomenological perspective, and it inspired the beginning of ubiquitous computing [27]. Furthermore, Mann [28] explained the use of smart glasses after over 35 years as a mediated version of reality. Those mediated relationships are reciprocal, and technology is perceived as the mediator between humans and the world. For example, a user looks *through* glasses rather than *at* glasses, and this is therefore termed as an embodiment relationship. In addition, smart glasses can represent information on a parallel screen while seeing the world, which is termed as a hermeneutic relationship [29]. A combined perspective of both adoption and mediation could complement each other in understanding the appropriation of smart glasses (Figure 2; derived and adapted from Zuidhof et al [30]). However, knowledge of the mediated experience of smart glasses is still scarce, and recent studies have reported strong influences of the social context in decision-making [17,21]. The theoretical framework of adoption and mediation can reveal both the user’s perspective and the perspective of others who are related to the user, such as a patient.

In the field of the philosophy of technology, mediation is interpreted as determining human-technology relationships and interactions, and anticipating influences on human behavior [29]. Prior studies on the adoption of smart glasses, as illustrated in Multimedia Appendix 1, not only present familiar constructs but also have begun, since 2010, to delve into social themes from various perspectives, including those of the user, conversational partner, and third parties [26]. A large-scale survey in which the anticipated perspective of the health care professional and the anticipated social interaction are united is lacking in current knowledge. Based on previous research results and the theoretical framework of adoption and mediation (as part of a doctoral research project), it seems relevant to explore adoption themes and assess if social interaction influences the intention to use smart glasses.

Figure 2 proposes to adapt the theoretical framework of adoption and mediation to the context of the health care professional and the patient. It is considered that smart glasses for health care professionals are still in an early phase of dissemination, and therefore, it is needed to gather knowledge about anticipating the use of smart glasses. Moreover, health care professionals are expected to anticipate adoption while taking into account social interaction with patients, but they can also take into account the patient’s perspective in social interaction. Therefore, this goes beyond mere adoption for self-interest and may lead to a higher or lower intention to use smart glasses.

### Objectives and Hypotheses

Prior research on the adoption of smart glasses has been mainly conducted in general contexts; however, some studies were carried out in the area of tourism or health care. Few of the recent qualitative studies on smart glasses in health care are part of the authors’ doctoral research projects. Furthermore, it appears that there are no large-scale surveys conducted on the adoption of smart glasses and that most of the recent studies have an explorative and qualitative nature. Prior research on the adoption of smart glasses is presented in Multimedia Appendix 1. In addition, the emotional and ethical aspects that can play roles as issues to adoption described above are still involved to a limited extent in recent studies and might be especially relevant in professions where people are vulnerable or interact intensively like in health care.

The objectives of this study were two-fold: (1) to develop an instrument that combines the perspectives of adoption and mediation to measure the intention to use smart glasses by health care professionals, and (2) to obtain insights into how the intention to use is influenced by adoption and social interaction aspects. Regarding the state of the art and the objectives of this study, the following research question was formulated: “To what extent do constructs derived from a mediation perspective contribute to the established constructs of adoption to explain the intention to use smart glasses?”

#### Observed Variables Derived From Technology Acceptance Literature

**Figure 3:**
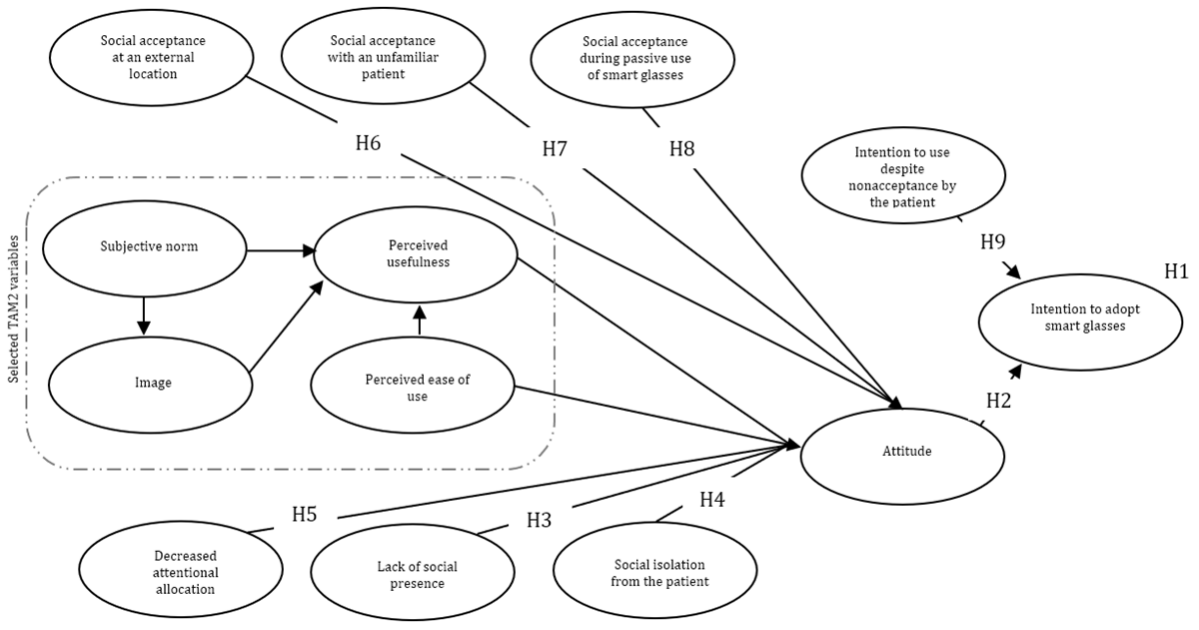
Conceptual model with the hypotheses of the anticipated adoption and social interaction of smart glasses by health care professionals. H: hypothesis; TAM: Technology Acceptance Model.

In the proposed model (Figure 2), it was argued that technology adoption and social influence might influence each other and the user. The underlying idea here is that a decision to use smart glasses is explained by a linear model and is not only aimed at the self but also others, as is endorsed by the mediation perspective [30,31]. The recently developed conceptual model to explain the anticipated adoption and the social aspects of smart glasses has been outlined in Figure 3, and the aspects will be included.

#### Observed Variables Derived From the Technology Acceptance Model 2

As one of the most accepted theories by scholars, in the TAM [13,14], intention to use is proposed as a key independent variable. The constructs “perceived usefulness” and “perceived ease of use” are mainly aimed at their considerations and do not yet tell us much about how this relates to others. Therefore, we found the Technology Acceptance Model 2 (TAM2) to be the most appropriate because it builds on the basic model and extends this model with constructs that take others into account, such as the constructs “subjective norm” and “image.” Other constructs from the TAM2 model, such as experience, voluntariness, job relevance, output quality, and result demonstrability, have been excluded as they extend beyond the scope of this study. These constructs do not specifically address social interaction, which is the central emphasis of our research. By adding specifically more social constructs, we aim to gain a more holistic perspective of not only personal considerations in own use but also how much a health care professional takes patients into account and how this influences the anticipated use of technology.

Hypothesis 1 is as follows: Additional social interaction constructs can explain the higher variance of users’ anticipated intention to use smart glasses compared with subjective norm, image, perceived usefulness, and perceived usefulness on anticipated intention to use.

The attitude toward technology is a debatable construct in adoption studies because the added value in the model might not be good enough [10], or it may theoretically differ little from the intention to use. Similar to the Theory of Planned Behavior [32], we regard attitude as a predictor of the intention to use smart glasses, and we added the construct “attitude” because of previous findings [31,33], which may be related to the early phase of the adoption of smart glasses where we can only measure the anticipated intention to use.

Hypothesis 2 is as follows: Users’ attitudes directly influence the anticipated intention to use smart glasses.

#### Observed Variables Regarding Social Interaction With Smart Glasses

Previous studies found several constructs under the themes that seemed theoretically relevant, such as attentional shift, social presence, and social isolation [14,31,34-36]. The items were translated as accurately as possible and applied to the anticipation of smart glasses. This resulted in the following constructs: lack of social presence, social isolation of the patient, and reduced attentional allocation to the patient.

Hypothesis 3 is as follows: The lack of social presence due to wearing smart glasses during a physical contact moment influences the attitude directly.

Hypothesis 4 is as follows: Social isolation due to wearing smart glasses during a physical contact moment influences the attitude directly.

Hypothesis 5 is as follows: The decreased attentional allocation due to wearing smart glasses during a physical contact moment influences the attitude directly.

#### Observed Variables Regarding Social Scenarios With Smart Glasses in Health Care

Furthermore, in previous qualitative studies, themes related to the context of smart glass use were also found, such as location, an unknown patient, and wearing smart glasses but not actively using them [31]. These scenarios were linked to social presence [36], resulting in social presence in 3 scenarios, namely, providing care with smart glasses on in an external location (outpatient care), providing care with smart glasses while in passive use, and providing care with smart glasses to an unfamiliar patient.

Hypothesis 6 is as follows: The use of smart glasses at an external location during a physical contact moment directly influences the attitude.

Hypothesis 7 is as follows: The use of smart glasses with an unknown person during a physical contact moment directly influences the attitude.

Hypothesis 8 is as follows: The passive use of smart glasses during a physical contact moment directly influences the attitude.

#### Observed Variables Regarding Nonacceptance of Smart Glasses by the Patient

The mediation approach visualizes the relationship with the outside world during adoption, while technology acts as a mediator in the work of the health care professional and the patient. To understand both the social interaction with patients during the use of smart glasses and the sustained use of smart glasses, the unintended consequences of smart glass use might also be relevant [26,37-39]. In particular, the patient’s nonacceptance of the use of smart glasses is interesting, simply because the patient’s acceptance of smart glasses would probably not create tension in the intentional use of smart glasses. The mediation perspective has led to the following construct in the model: intention to use despite social nonacceptance by others.

Hypothesis 9 is as follows: The intention to use smart glasses despite the nonacceptance of smart glasses by the patient directly influences the anticipated intention to use.

## Methods

### Study Design

To achieve the aims of this study, a summated rating scale development approach for social sciences was used [40]. The initial steps were aimed at defining constructs and designing the scale. The resulting scale was evaluated through an online survey among a sample of students.

### Defining Constructs and Item Pool Development

To develop an item pool, existing literature was consulted, and the state-of-the-art knowledge, as described in the Introduction, was combined with the theoretical framework of adoption and mediation. A core aspect of the framework (Figure 2) is the reciprocal relationship among the user, the technology, and the world. In the next step, the aspects from Figure 2 were substantiated with existing literature and translated into items. See Multimedia Appendix 2 for the item pool. All measurements were recorded using 5-point Likert scales. Back translation was performed by 2 researchers as a validation method for the survey [41] and to develop a robust item pool.

### Item Pool Refinement

To check for comprehensibility, readability, and internal consistency, 2 pretest moments were organized. First, the questions were reviewed by 2 potential respondents. Next, data collection (as a pretest) was conducted at a health care institution in the Netherlands (n=17). Although the pretest was conducted on a small sample, internal consistency was acceptable. Initially, reversed items were included, but inconsistent response patterns were observed for those items. Respondents reported too much confusion with reversed items, and therefore, these items were removed from the questionnaire.

### Data Collection

Respondents (N=450) were recruited in July 2022 on the internet using Amazon’s Mechanical Turk. The questionnaire was made available to respondents who met the following inclusion criteria: (1) language: English; (2) employment industry: health care; and (3) location: Northwest Europe (Austria, Belgium, Denmark, Finland, France, Germany, Iceland, Ireland, Luxembourg, Netherlands, Norway, Sweden, and Switzerland), Canada, United Kingdom, and United States. The questionnaire was closed once the desired number of respondents was reached.

### Demographic Information

A summary of the demographic information of the respondents is presented in Table 1. The sample pool consisted mainly of male participants, with over three-quarters being 42 years of age or younger. The majority of the respondents were from the United States. With regard to occupation, the participants were distributed as follows: physicians, 192 (42.6%); nurses, 165 (36.7%); and others, 93 (20.7%). Other professions included a diverse group of medical assistants (n=14), medical technical professionals (n=11), managers (n=10), health care professionals (n=9), IT professionals (n=6), and home care professionals (n=4), among other various professions in health care.

**Table 1:** Demographic information of the respondents.

Characteristic		Value (N=450), n (%)
**Gender**		
	Female	303 (32.4)
	Male	146 (67.3)
	Prefer not to say	1 (0.2)
**Age (years)**		
	>72	1 (0.2)
	63-72	15 (3.3)
	53-62	42 (9.3)
	43-52	45 (10.0)
	33-42	124 (27.6)
	23-32	222 (49.3)
	18-22	1 (0.2)
**Country of residence**		
	Canada	4 (0.9)
	United States of America	442 (98.2)
	Northwest Europe	3 (0.7)
	United Kingdom	1 (0.2)
**Highest degree**		
	High school or less	5 (1.1)
	Some college but no degree	22 (4.9)
	Associate degree	32 (7.1)
	Bachelor’s degree	235 (52.2)
	Master’s degree	145 (32.2)
	Professional or doctoral degree	11 (2.4)
**Current profession**		
	Physician	192 (42.6)
	Nurse	165 (36.7)
	Other	93 (20.7)
**Employment status**		
	Employed full time	401 (89.1)
	Employed part-time	35 (7.8)
	Student	4 (0.9)
	Self-employed	10 (2.2)
**Years of work experience**		
	<10	224 (49.8)
	11-20	115 (25.6)
	21-30	74 (16.4)
	31-40	28 (6.2)
	>40	9 (2.0)

### Ethical Considerations

Before starting the survey, respondents provided active online consent after reading the opening statement (Multimedia Appendix 3). Furthermore, pictures were used with permission from health care professionals and patients. Respondents were rewarded with US $2 when they completed the survey. Study data were deidentified and anonymously used for analysis. This study was reviewed and approved by the Ethical Review Board of the University of Twente (approval number 211121).

### Procedure

**Figure 4:**
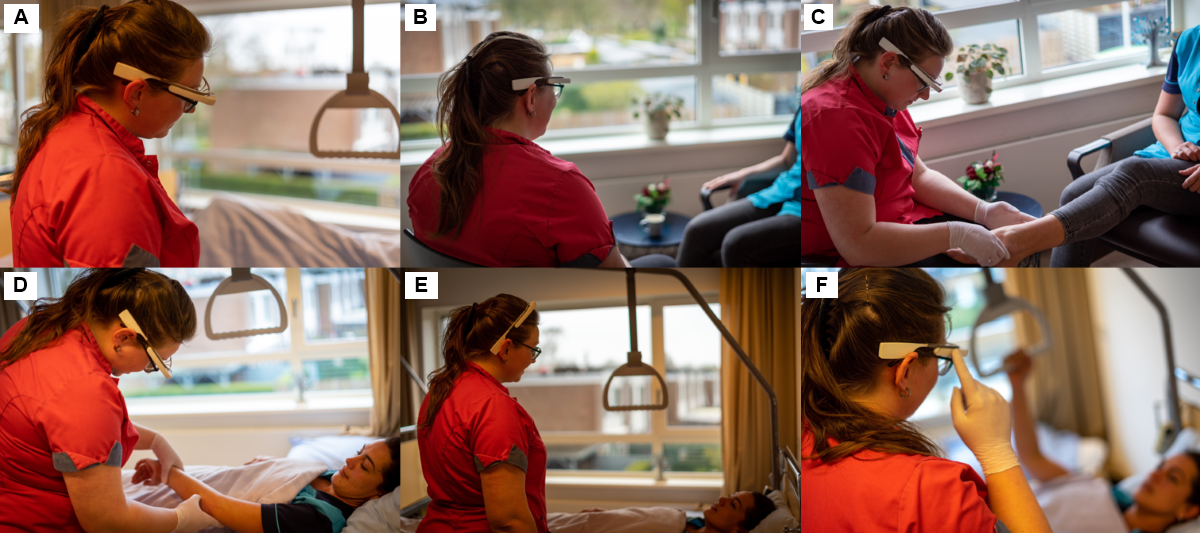
Photos used in the questionnaire about examples of using smart glasses consecutively: (A) contact with patients, (B, C) face-to-face interaction in outpatient care, (D) inpatient care, (E) passive use of smart glasses, and (F) active use of smart glasses (published with permission).

After the opening statement and consent, respondents were asked to watch 2 short videos about smart glasses to get an idea of the technology and its use. The first video showed an augmented reality platform for health care with English-spoken animations [42], and the second video showed an example of Vuzix M400 smart glasses in the context of health care [43]. After watching both videos, respondents were asked to fill out the questionnaire. Demographic information was requested at the end of the questionnaire. Some photos used in the questionnaire are presented in Figure 4.

### Data Analysis

Before the analyses, data were checked for normality. No significant deviation from normality was found. All skewness and kurtosis values were below 1.96, and values between –2 and +2 are considered acceptable to prove normal distribution [44]. Structural Equation Modeling was performed with maximum likelihood estimation, as advocated by [45,46], to test the conceptual model of the intention to adopt smart glasses by health care professionals. To test if the conceptual model fits the data well, the next absolute and incremental model fit indices were used as suggested by Holbert and Stephensen [47] and Hoyle and Panter [48]. The most common absolute fit index is the *χ^2^* goodness-of-fit test, and to minimize the impact of sample size on the model chi-square, the relative/normed chi-square (*χ^2^*^/df^) was used [49]. The standardized root mean square residual (SRMR) was reported as an absolute fit index. The following incremental fit indices have been reported: Tucker-Lewis index (TLI) and root mean square error of approximation (RMSEA). The practitioner recommendations for model evaluation were followed, suggesting a cutoff value close to 0.95 for the TLI in combination with a cutoff value close to 0.09 for the SRMR to evaluate model fit, and a cutoff value close to 0.06 for the RMSEA [50]. The Fornell and Larcker discriminant validity criterion was used to test discriminant validity. The Fornell and Larcker criterion is satisfied when a construct is more closely related to its own indicators than to other constructs [51].

## Results

### Diffusion of Smart Glasses in Health Care

The questionnaire on the anticipated adoption and mediation of smart glasses was designed to examine the perceptions of future users regarding smart glasses. It was also designed to get a better understanding of how specific situations in the use of smart glasses influence the intention to use them. The research question was: “To what extent do constructs derived from a mediation perspective contribute to the established constructs of adoption to explain the intention to use smart glasses?”

Participants were queried regarding their familiarity and experience with smart glasses. The constructs “knowledge” and “persuasion” served as constructs in assessing how health care professionals perceive the diffusion of smart glasses. Overall, the respondents reported having substantial knowledge about smart glasses (eg, I know what someone can do with smart glasses; mean score 4.02, SD 0.84) and expressed understanding of the concept of smart glasses (mean score 4.08, SD 0.79). Close to 75% of the respondents (n=339) indicated their willingness to try smart glasses if they were available to them (mean score 3.94, SD 0.93).

### Explaining Anticipated Adoption of Smart Glasses: Model Comparison

To investigate the added value of the observed variables in the conceptual model, a comparison was made between validated TAM2 [52] constructs used to create a baseline and the additional social interaction variables in this study. The constructs “perceived usefulness” and “perceived ease of use” were adapted to the context of smart glasses, and subjective norm and image were included because they represent social interaction. Although the construct “attitude” was not included in the TAM, there was a reason to include this validated construct based on previous studies on the early phase of the diffusion of smart glasses.

### Interpreting Selected TAM2 Effects: Intention to Use, Perceived Usefulness, Perceived Ease of Use, Subjective Norm, and Image

#### Measurement Model

The initial measurement model generated a good fit (*χ^2^*_142_=183.55; *χ^2^*^/df^=1.29; SRMR=0.03; TLI=0.99; RMSEA=0.03 [CI 0.01-0.04]). The internal consistency was above the aspirational level (α>.70).

#### Structural Model

The results obtained from testing the validity of a causal structure of earlier validated variables showed a good fit (*χ^2^*_14_=218.314; *χ^2^*^/df^=1.52; SRMR=0.04; TLI=0.98; RMSEA=0.03 [CI 0.02-0.04]).

#### Path Model

**Figure 5:**
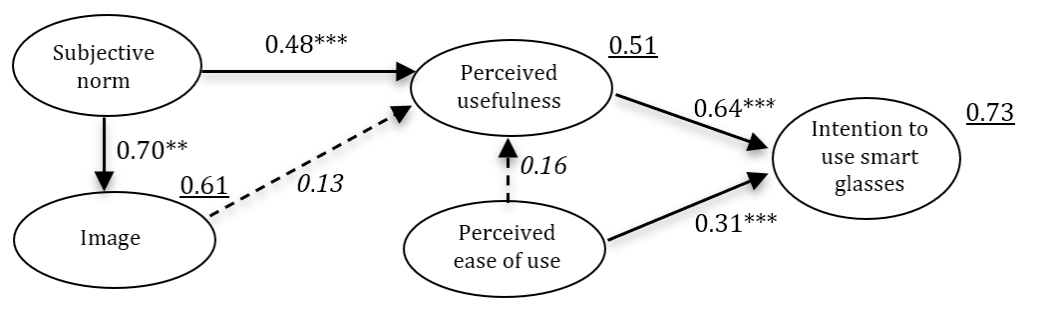
Standardized path coefficients of the TAM2 variables for anticipated use of smart glasses by health care professionals (N=450). Dotted lines represent nonsignificant paths, italics represents nonsignificant factor loadings, and underline represents squared multiple correlations. TAM: Technology Acceptance Model. ***P<.001.

The path model with standardized path coefficients is featured in Figure 5. The standardized path coefficients show significant direct effects of perceived usefulness and perceived ease of use on the intention to use smart glasses. Furthermore, subjective norm had a direct effect on perceived usefulness. Figure 5 also shows that subjective norm has a direct effect on image. The direct effect of perceived ease of use on perceived usefulness was not significant. The direct effect of image on perceived usefulness was also not significant. Subjective norm was the only contributor to perceived usefulness. In addition, perceived usefulness was the strongest contributor to the intention to use smart glasses. Squared multiple correlations showed that image accounted for 61%, perceived usefulness accounted for 51%, and intention to use smart glasses accounted for 73% of the explained variance.

### Interpreting Social Interaction Effects: Lack of Social Presence, Social Isolation, Decreased Attentional Allocation, External Location, Unfamiliar Person, Passive Use, Intention to Use Despite Nonacceptance by Others

#### Measurement Model

The initial measurement model generated a good fit (*χ^2^*_1461_=2146.696; *χ^2^*^/df^=1.47; SRMR=0.04; TLI=0.95; RMSEA=0.03 [CI 0.03-0.04]). The internal consistency was above the aspirational level (α>.70).

#### Structural Model

The results obtained from testing the validity of a causal structure of the conceptual model showed that the initial model did not fit the data (*χ^2^*_1525_=4896.900; *χ^2^*^/df^=3.21; SRMR=0.25; TLI=0.78; RMSEA=0.07 [CI 0.07-0.07]). Post-hoc modification indices suggested an improved fit by correlating error terms of perceived ease of use and social norm, social norm and image, lack of social presence and decreased attentional allocation, lack of social presence and social isolation, decreased attentional allocation and intention to use, external location and passive use, unfamiliar person and passive use, passive use and social norm, and passive use and intention to use despite social nonacceptance by others. Furthermore, post-hoc indication indices also suggested additional paths between perceived usefulness and intention to use, perceived ease of use and intention to use, perceived ease of use and image, perceived ease of use and social isolation, social norm and external location, social norm and lack of social presence, and lack of social presence and intention to use despite social nonacceptance by others. Some of the paths in the conceptual model were suggested differently, such as external location to intention to use instead of attitude, and the same applied for unfamiliar people and passive use. The paths suggested were from attitude to intention to use despite social nonacceptance by others and attitude to lack of social presence. The respecified model generated a good fit (*χ^2^*_1337_=2113.035; *χ^2^*^/df^=1.58; SRMR=0.08; TLI=0.95; RMSEA=0.04 [CI 0.03-0.04]). Multimedia Appendix 2 summarizes the mean and SD, Cronbach α, factor loadings (β), and squared multiple correlations (*R^2^*) of the observed variables to predict the anticipated intention to use smart glasses. The correlation matrix is available upon request.

#### Path Model

**Figure 6:**
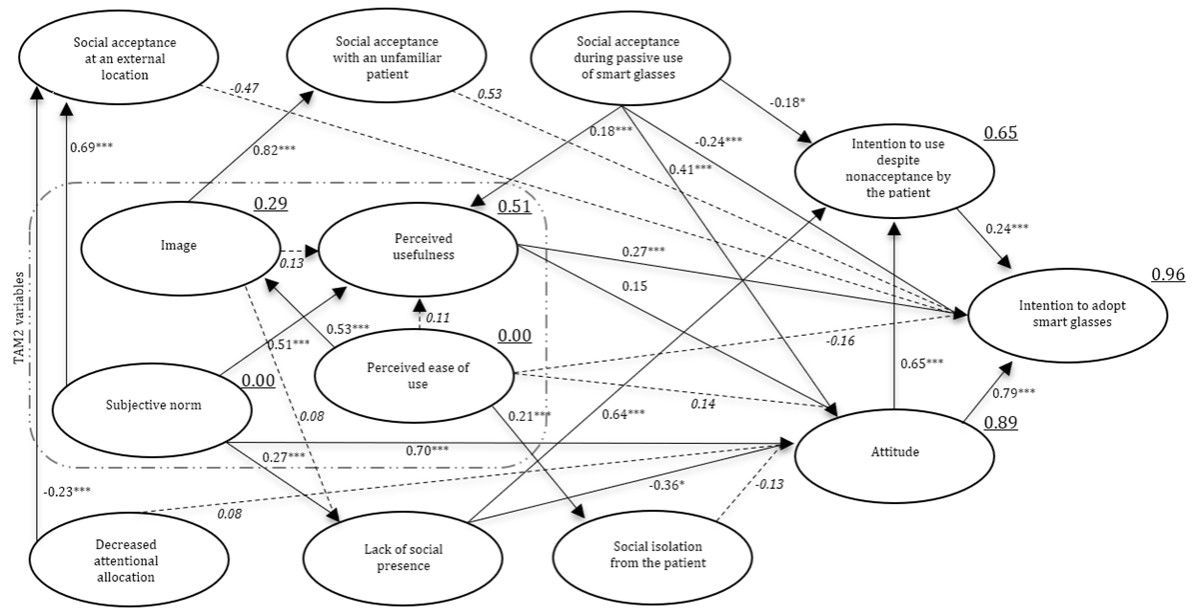
Standardized path coefficients of the adoption of smart glasses and the social interaction variables for the anticipated use of smart glasses by health care professionals (N=450). Dotted lines represent nonsignificant paths, italics represents nonsignificant factor loadings, and underline represents squared multiple correlations. TAM: Technology Acceptance Model. *P<.05; ***P<.001.

It was hypothesized that additional social interaction constructs could explain higher variance of users’ anticipated intention to use compared to subjective norm, image, perceived usefulness, and perceived usefulness on anticipated intention to use (hypothesis 1). Squared multiple correlations are featured in Figure 6. It was shown that intention to use represented 96%, and it was raised with additional social interaction variables compared with squared multiple correlations, with representation of 73% from intention to use, perceived usefulness, perceived ease of use, subjective norm, and image. Attitude accounted for 89%, intention to use smart glasses despite social nonacceptance accounted for 65%, perceived usefulness accounted for 51%, and image accounted for 29%. These results supported the acceptance of hypothesis 1.

It was hypothesized that the user’s attitudes directly influence the anticipated intention to use smart glasses (hypothesis 2). The standardized path coefficients in Figure 6 show a significant direct effect of attitude toward smart glasses on the intention to use smart glasses, which supported the acceptance of hypothesis 2.

It was stated that the lack of social presence due to wearing smart glasses during a physical contact moment influences the attitude directly (hypothesis 3). Figure 6 shows a significant negative direct effect of lack of social presence on attitude, and therefore, the results supported the acceptance of hypothesis 3. Surprisingly, the lack of social presence also had a positive direct effect on the intention to use despite social nonacceptance by patients.

Furthermore, it was hypothesized that social isolation due to wearing smart glasses during a physical contact moment influences the attitude directly. Social isolation did not have a significant effect on attitude (Figure 6), and this result supported the rejection of hypothesis 4.

In addition, it was hypothesized that the decreased attentional allocation due to wearing smart glasses during a physical contact moment influences the attitude directly (hypothesis 5). Figure 6 shows that decreased attentional allocation does not have a significant effect on attitude, and this result supported the rejection of hypothesis 5.

It was also hypothesized that the social acceptance of the use of smart glasses at an external location during a physical contact moment directly influences attitude (hypothesis 6). Figure 6 shows that use at an external location could not significantly explain attitude, and this result supported the rejection of hypothesis 6.

In addition, it was hypothesized that social acceptance regarding the use of smart glasses with an unknown person during a physical contact moment directly influences attitude. Figure 6 shows that there was no significant effect of the use of smart glasses with an unknown person on attitude, and this result supported the rejection of hypothesis 7.

Furthermore, it was hypothesized that the passive use of smart glasses during a physical contact moment directly influences attitude. Figure 6 shows that social acceptance regarding the passive use of smart glasses indeed directly influences attitude. Therefore, hypothesis 8 is accepted. In addition, social acceptance regarding the passive use of smart glasses has significant effects on the intention to use despite social nonacceptance by the patient, intention to use, and perceived usefulness.

**Figure 7:**
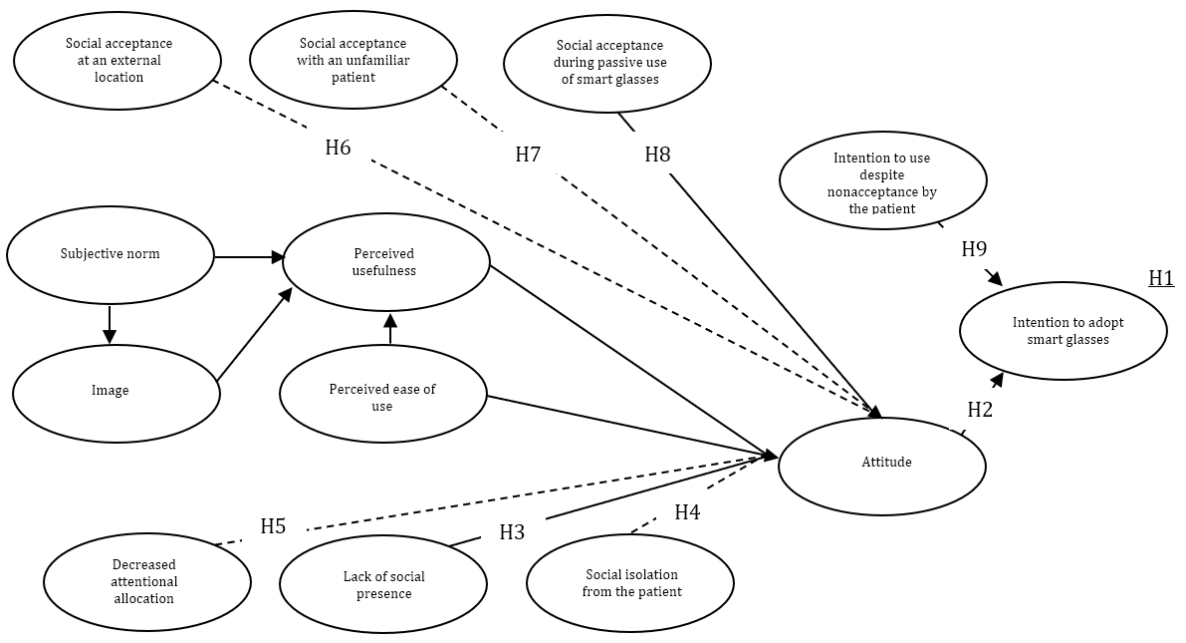
Overview of the tested hypotheses of the adoption of smart glasses and the social interaction variables for the anticipated use of smart glasses by health care professionals (N=450). Hypothesis 1 is underlined, and the hypothesis is accepted. Solid lines represent accepted hypotheses, and dotted lines represent rejected hypotheses. H: hypothesis.

Examining the nonacceptance of smart glasses by the patient, it was hypothesized that the intention to use despite the nonacceptance of smart glasses by the patient directly influences the anticipated intention to use (hypothesis 9). Figure 6 shows a significant effect, and therefore, hypothesis 9 is accepted. In addition, an overview of the accepted and rejected hypotheses can be found in Figure 7.

## Discussion

### Overview

Considering the current state of the art and the objectives of this study, we formulated the following research question: “To what extent do constructs derived from a mediation perspective contribute to the established constructs of adoption to explain the intention to use smart glasses?” This study had 2 primary objectives. The first objective was to develop an instrument that combines the perspectives of adoption and mediation to measure the intention to use smart glasses by health care professionals. A conceptual model was created by drawing on previous findings related to smart glass adoption and testing hypotheses about their anticipated effects on attitudes and intentions to use. The instrument developed integrates both adoption and social interaction perspectives, highlighting smart glasses as a mediating factor between individuals, and it was validated with health care professionals. The approach for this instrument builds on prior research and represents a step forward in understanding the role of smart glasses in health care.

The second objective was to obtain insights into how the intention to use is influenced by adoption and social interaction aspects. The structural equation analysis showed that integrating social interaction factors with selected TAM2 constructs led to a higher explained variance in the intention to use smart glasses than TAM2 alone. Key factors influencing the intention to use included attitude, perceived usefulness, intention to use despite social nonacceptance, and passive use of smart glasses. Social presence had an unexpected effect: as social presence increased, attitudes toward smart glasses decreased. Despite social nonacceptance by patients, health care professionals still showed willingness to use smart glasses, suggesting that this factor may be less decisive in health care. Passive use was linked to perceived usefulness and intention to use, indicating that wearing smart glasses on top of the head (as presented in Figure 4) could be convenient to have smart glasses close by, even when not actively in use. These findings suggest that social interaction plays a nuanced role in adoption, and further research is recommended to explore these relationships, especially regarding social presence and passive use.

### Principal Findings

#### Development of the Instrument

Regarding the first objective of this study, there was an initial endeavor to create an instrument for investigating the anticipated adoption and mediation (as expressed through social interactions) of smart glasses within the health care sector. Consequently, a conceptual model was derived, which (1) drew upon prior findings related to the adoption of smart glasses, (2) tested hypotheses pertaining to the anticipated effects on attitudes and use intention, and (3) was applied to a sample of health care professionals for validation. We believe that the proposed conceptual model of expected adoption and social interaction provides a strong theoretical basis for further development of the model. The structural model in this study adds a higher explained variance but can be improved in the parsimony of the model. Furthermore, the focus in the model can be shifted in later research studies to other bystanders, such as colleagues, who may be confronted with a user of smart glasses.

The initial objective of this research was to develop an instrument that integrates the perspectives of both adoption and mediation. The resultant instrument was crafted to align closely with the state-of-the-art knowledge of smart glass adoption while simultaneously addressing aspects of social interaction. Central to this development was the assumption that smart glasses serve as a mediating factor between individuals. While there exist numerous methods to investigate social interaction factors, this study opted to ground its approach in findings from previous research.

### Explaining the Anticipated Adoption of Smart Glasses

The second aim of this study was to gain insights into how the intention to use is influenced by adoption and social interaction aspects. The outcomes of the structural equation analysis revealed that our path model (Figure 6) integrating constructs from both TAM2 and social interaction variables demonstrated a higher explained variance in the anticipated intention to use smart glasses, compared to the isolated TAM2 constructs that were selected. This finding indicates that social interaction concerning smart glasses plays a role in anticipating the adoption of smart glasses in health care besides the perceived usefulness and attitude toward smart glasses. More specifically, the intention to use smart glasses is directly influenced by attitude, perceived usefulness, intention to use despite social nonacceptance, and passive use of smart glasses.

The presented model showed that attitude and intention to use both had high squared multiple correlations. This gives a reason to consider whether there is a theoretical difference between these constructs. In the Introduction section, it was also indicated that the attitude toward technology is a debatable construct in adoption studies because the added value in the model would not be substantial [10]. Original adoption models were developed for computer use in the 1980s, but smart glasses are different in several ways: they are worn on the body, are in the field of view, and often contain a camera. In this model, attitude is a mediating variable for subjective norm and the lack of social presence and was found to be important for the intention to use. Follow-up studies may investigate this relationship further.

Intention to use despite social nonacceptance shows that the influence of others is important for the health care professional but that it is not decisive to eventually use smart glasses. This can be understood because care actions can often be unpleasant for the patient but necessary for health purposes. This construct may score very differently in other disciplines where social relationships may be more important. Furthermore, the anticipated intention to use smart glasses is indirectly influenced by the lack of social presence and subjective norm.

A notable result of the lack of social presence is the decline in attitude. If the lack of social presence decreases (with an increase in social presence), the attitude toward smart glasses also decreases. This might indicate that respondents do not expect that smart glasses will ever provide more social presence and would not use them for that reason. One explanation that supports this finding is the positive regression coefficient with intention to use despite social nonacceptance by patients. This suggests that professionals would use smart glasses despite social nonacceptance by the patient.

The results of this study also indicate that there is a complex relationship between the passive use of smart glasses and 4 variables: a direct relationship with intention to use and an indirect relationship through perceived usefulness, attitude, and intention to use despite social nonacceptance. This can have 4 explanations, namely that passive use has a negative relationship with the intention to use, which indicates, for example, that health care professionals would rather not passively use smart glasses. However, this seems to contradict the finding of a weaker relationship with perceived usefulness, which could mean, for example, that it is convenient to have the glasses quickly at hand by wearing them on top of the head, just as a doctor places a stethoscope on the neck. Passive use also leads to an increased positive attitude toward glasses, which in turn leads to increased intention. In addition, there was a significant negative regression between passive use and intention to use despite social nonacceptance by the patient. This could indicate, for example, that the health care professional is willing not to use technology or, in this case, is willing to remove it if the smart glasses are not actively used and the patient is not comfortable with them.

The second objective was to gain insights into how the intention to use smart glasses is influenced by the aspects of both adoption and social interaction. Based on our findings, it can be asserted that the presented model provides a higher explained variance for the intention to use smart glasses. The majority of the hypotheses were confirmed. However, some results were unexpected, such as the influence of social presence on attitudes, as discussed previously. Contrary to expectations drawn from prior research [31,53], we found that a lack of social presence did not result in a negative attitude or diminished intention to use. Therefore, we recommend further exploration in subsequent research, potentially using methodologies such as in-depth interviews and quasiexperimental studies. The same applies to the construct of social acceptance during the passive use of smart glasses. The model indicated that this factor also impacts perceived usefulness, suggesting that passive use of smart glasses could be beneficial in a work context and could consequently enhance the intention to use. Further research should shed more light on the underlying reasons for this finding.

### Limitations

This study focused on the combination of adoption and social interaction. A limitation of this study lies in the comparison with the full TAM2 model. A priori–selected TAM2 constructs based on relevance and the aim of the study were included, namely to investigate social interaction and assess to what extent themes from the mediation perspective add something to current models. A comparison with the full TAM2 model is recommended for a follow-up study.

Second, in this study, social acceptance was examined through scenarios and not directly as a variable. The reason for this was to tie in with the previous findings and become more concrete in the specific context. This ignores social acceptance as an independent construct, and it is advisable to do so now that it appears that social interaction plays a substantial role in the model.

Third, as discussed in the conclusion, attitude and intention to use as variables score high on the explained variance, and it is questionable whether they are distinctive enough. Nevertheless, this difference is based on previous literature. Attitude was assumed as a preliminary state in the conceptual model, and it also fits the final model. It is recommended to investigate this further in a subsequent study, and it might be related to the last limitation.

The sample was limited to individuals who have access to MTurk. The distribution of respondents was mainly centered in the United States, and the questionnaire may have been completed mainly by people who were enthusiastic about the topic. As other researchers have addressed potential issues with sample representativeness and quality of the data [54,55], the results are more difficult to generalize and are not necessarily representative of the population of health care professionals. Therefore, it is important to revalidate the results with a reliable and representative dataset.

Furthermore, participants were presented with 2 videos to watch before proceeding further with the questionnaire. These videos, accessible to the public, were specifically chosen to provide a balanced depiction of what smart glasses are and their capabilities. It is worth noting, however, that as commercial videos, they may be designed to persuade viewers of the utility of smart glasses. The researchers selected these videos because they provided examples that were realistic and applicable in real-life work contexts at the time of the study and represented an attempt to prevent unrealistic imaging from smart glasses.

Although this model has been created based on previous literature and has remained as close as possible to the conceptual model, it is not a parsimonious model. From Cutting [56], a series of criteria can be derived to discuss the model evaluation, which is reflected by the accuracy, scope, and simplicity of the model. Parameter counting is widely used to indicate simplicity: if 2 models are both equally valid, the one with the fewest parameters is the best [56]. Several nonsignificant regression coefficients were found in the results of this study, and in combination with the previous recommendations, a subsequent simpler model can be developed.

### Conclusions

#### Anticipating Adoption and Appropriation

The main finding is the answer to the following research question: “To what extent do constructs derived from a mediation perspective contribute to the established constructs of adoption to explain the intention to use smart glasses?” In the Introduction, the assumption was made that the target audience would likely be in an early stage of adoption of smart glasses. However, the responses gathered concerning knowledge and experience revealed that the majority of respondents demonstrated a fair understanding of smart glasses, and approximately half of them had already experimented with the technology.

The mediation perspective has provided insights into potentially useful variables for the anticipated adoption and social interaction of smart glasses. Social interaction with technology may be important because smart glasses are worn on the body. Venkatesh and Davis [52] found that the influence of subjective norm was only significant in mandatory usage contexts and not in voluntary contexts [52]. In this study, no obligatory or voluntary context was specifically referred to, yet subjective norm had a significant association with intention to use. That moderator can be added for control in a subsequent study, but it is also expected that with the current technology, such as smart glasses that are worn on the body, this is different than with personal computers. With smart glasses, there is human interaction where technology differently mediates in the physical world. In addition, social influences would have a nonsignificant influence on intention to use without moderators such as gender, age, experience, and use voluntariness [10]. This study showed that subjective norm and the lack of social presence influence attitude and indirectly influence intention to use smart glasses in health care and can therefore make better predictions for anticipated use intention for smart glasses. Furthermore, despite many of the social issues addressed in the literature [21,26,31] and previous studies, most of the issues incorporated in this study did not have an effect on the anticipated intention to use smart glasses in the future. The findings reported here shed new light on the intention to use smart glasses in health care, where it could be argued that social interaction is perceived as an important aspect in providing care but not the most important construct for predicting future use. While this study does not provide evidence regarding the issues in social interaction surrounding smart glasses in health care as seen in the literature, a limitation in this study might be that respondents did not experience the use of smart glasses in real time. On the other hand, it may also be that the quality of care is more important than social interaction, and even if a patient feels uncomfortable when it comes to health, an intervention is sometimes necessary. Respondents might assume that smart glasses will help them in their work and that they will add value, regardless of the outcomes of social interaction.

Furthermore, although it is suggested that social acceptance is regularly included in evaluation sections in smart glasses research [18,57], it was observed that the adoption was only partly included in research designs in some studies. For example, in some cases, technology acceptance was evaluated by 3 constructs: perceived usefulness, perceived ease of use, and intention to use [58]. However, the first TAM [11] included attitude as well, and the more recent developments of the model included 13 factors influencing perceived usefulness and perceived ease of use and thereafter intention to use [15,59]. This example and current research in technology acceptance in related fields [60,61] show that there is more to study in adoption than 3 factors, and we argue to use all relevant aspects of adoption and acceptance frameworks to obtain more valid answers in acceptance research.

In summary, several theoretical perspectives have been used in this study, and it has been found that despite the concerns raised about social interaction with smart glasses, these aspects play important roles for health care professionals but are ultimately not decisive for the intention to use. As a result, there are fewer threats to implementation for health care than might be expected based on previous literature. It can also be concluded that the empirical findings of this study provide a new understanding of how the mediation perspective can increase the explained variance compared to existing constructs from the literature. This is why the mediation perspective adds new insights into a theoretical framework that can also be applied in other professional fields or related technologies.

## Appendix

Multimedia Appendix 1 Prior research on the adoption of smart glasses.

Multimedia Appendix 2 Item pool, descriptive statistics, factor loadings, squared multiple correlations, and Cronbach alpha of the observed indicators to explain and predict the anticipated use of smart glasses.

Multimedia Appendix 3 Opening statement.

## Abbreviations

RMSEA: root mean square error of approximation
SRMR: standardized root mean square residual
TAM: Technology Acceptance Model
TAM2: Technology Acceptance Model 2
TLI: Tucker-Lewis Index
UTAUT: Unified Theory of Acceptance and Use of Technology

## Data Availability

The datasets generated during or analyzed during this study are available from the corresponding author upon reasonable request.
