# Supplementary material for: Social Acceptance of Smart Glasses in Health Care: Model Evaluation Study of Anticipated Adoption and Social Interaction
Source: JMIR Form Res. 2025 Feb 11;9:e49610. doi: 10.2196/49610 (PMC11862762; doi:10.2196/49610)
Supplement: Multimedia Appendix 1 [file formative_v9i1e49610_app1.docx]

**Multimedia Appendix 1.** Prior research on the adoption of smart glasses.

| **Reference** | **Context** | **Methods and sample** | **Findings** |
| --- | --- | --- | --- |
| [1] | Researchers with different areas of expertise | Qualitative focus groups (*n* = 7) quantitative, user study (*n* = 38) | Social context (interpersonal communication) influences usage norms (more critical), public use is controversial (freedom of choice in own use vs. privacy protection third parties), privacy violation by data glasses, knowledge about usage/performed actions by others, and expectations about recording. |
| [2] | Randomly invited German respondents | Quantitative, survey, (*n* = 146) | Open and emotionally stable consumers are more aware of Google Glass. Perceived functional benefits and social conformity of smart glasses are more likely to adopt. |
| [3] | Physicians | Interviews (*n* = 8), expert focus group (*n* = 7), experimental study (*n* = 75). | Compatibility, ease of reminding, speech recognition, ease of use influence usefulness positively. Ease of learning, ease of medical education, external influence and privacy affect ease of use positively. |
| [4] | Individuals from the United States | Survey, *n* = 1200 | Head-mounted displays were perceived more acceptable if used to support a person with a disability. |
| [5] | Individuals in Germany | Multiple-year case study, *n* = 118 | No significant change toward a positive attitude between 2014 and 2016. Utility and usability are more valued for long-term adoption compared to social acceptability. Unobtrusive design could improve social image. |
| [6] | Cultural heritage tourists | Proposed model | External variables such as information quality, technology readiness, visual appeal, and facilitating conditions influence beliefs, attitude, and usage intention. |
| [7] | Students | Quantitative, Online survey, *n* = 228 | Life efficiency, enjoyment, reality enhancement, socializing, self-expression drive ARSG usage intention. |
| [8] | Students, North America | Online survey, *n* = 285 | Expected utilitarian, hedonic, and symbolic benefits drive consumers’ reactions. Other people’s privacy can strongly influence users’ decision-making. |
| [9] | British female tourists | Focus groups, *n* = 44 | Dimensions found were information quality, system quality, costs of use, recommendations, personal innovativeness, risks, and facilitating conditions. |
| [10] | N/A | Proposed model | Integration of TAM, DOI, and Rauschnabel. |
| [11] | Tourism | Qualitative, Interviews, Thematic analysis, *n* = 28 | Personal innovativeness seemed not dominant, personal differences influence ease of use, obtrusiveness. Perceived usefulness and perceived enjoyment were perceived as benefits. Privacy, risk of use, and cost were obstacles to adoption. Social aspects are a disturbance to people, and limited social interaction with others. |
| [12] |  | Observations | Participants wearing CareLenses felt uncomfortable interacting with patients: feeling strange, and ridiculous. Could be overcome by wearing all HMDs together (equality), or by letting others also see what the user sees (transparency). |
| [13] | YouTube commenters | Qualitative, content analysis, *n* = 124 | Positive attitude was found for gaming, coolness, and perceived need. Negative attitudes were expressed by judgments, emotions, and comparisons to other products. |
| [14] | Human resources, industry | Qualitative, Focus groups, *n* = 63 | TAM2 constructs were confirmed, and Data protection, strategic value, and systematic communication were perceived as influential aspects. |
| [15] | Retailing | Quantitative, SEM, *n* = 126 | Perceived enjoyment functions as a direct predictor of attitude. |
| [16] | Healthcare professionals | Quantitative, PLS-SEM, *n* = 119 | Documentation affects intention. Integrations with IS affect perceived usefulness. Technological compatibility, external factors, and hands-free feature explain perceived ease of use. Other factors hinder people to adopt. |
| [17] | Agricultural domain | User test, *n* = 7 | Comfort issues like harm and disturbance of the visual field. Voice mode left poorer objective and subjective evaluations compared with buttons. |
| [18] | Neuro-  interventionalists | User test, *n* = 5 | Comfortable to wear, except with prescription glasses. No complex installation and thus accessible. Remote controlling the camera can be difficult due to its fixed view. |
| [19] | Healthcare | Qualitative, content analysis, *n* = 26 | Themes related to anticipated adoption are foreknowledge, innovativeness, use cases, ethical issues, and attitude. Themes related to social influences were from the anticipated use perspective: attention shift feels natural in work settings, uncomfortable in social situations. The reactions to the anticipated use by others were voiced by the need for a clear purpose and etiquette, social isolation, design, context of use, camera use, lack of control, and negative emotions. |
| This study |  |  | Proposing a multi-perspective adoption and mediation model for smart glasses |

References:

1. Koelle M, Kranz M, Möller A. Don’t look at me that way! - Understanding User Attitudes Towards Data Glasses Usage. MobileHCI 2015 - Proceedings of the 17th International Conference on Human-Computer Interaction with Mobile Devices and Services 2015;(August):362–372. doi: 10.1145/2785830.2785842

2. Rauschnabel PA, Brem A, Ivens BS. Who will buy smart glasses? Empirical results of two pre-market-entry studies on the role of personality in individual awareness and intended adoption of Google Glass wearables. Comput Human Behav Elsevier Ltd; 2015;49:635–647. doi: 10.1016/j.chb.2015.03.003

3. Goken M, Basoglu AN, Dabic M. Exploring adoption of smart glasses: Applications in medical industry. 2016 Portland International Conference on Management of Engineering and Technology (PICMET) IEEE; 2016. p. 3175–3184. doi: 10.1109/PICMET.2016.7806835

4. Profita H, Albaghli R, Findlater L, Jaeger P, Kane SK. The AT effect: How disability affects the perceived social acceptability of head-mounted display use. Conference on Human Factors in Computing Systems - Proceedings 2016;4884–4895. doi: 10.1145/2858036.2858130

5. Koelle M, el Ali A, Cobus V, Heuten W, Boll SCJ. All about acceptability? Identifying factors for the adoption of data glasses. Conference on Human Factors in Computing Systems - Proceedings 2017;2017-May:295–300. doi: 10.1145/3025453.3025749

6. Obeidy WK, Arshad H, Huang JY. An acceptance model for smart glasses based tourism augmented reality. AIP Conf Proc 2017;1891(October). doi: 10.1063/1.5005413

7. Rauschnabel PA. Virtually enhancing the real world with holograms: An exploration of expected gratifications of using augmented reality smart glasses. Psychol Mark 2018;35(8):557–572. doi: 10.1002/mar.21106

8. Rauschnabel PA, He J, Ro YK. Antecedents to the adoption of augmented reality smart glasses: A closer look at privacy risks. J Bus Res Elsevier; 2018;92(August):374–384. doi: 10.1016/j.jbusres.2018.08.008

9. tom Dieck MC, Jung T. A theoretical model of mobile augmented reality acceptance in urban heritage tourism. Current Issues in Tourism Routledge; 2018 Jan 22;21(2):154–174. doi: 10.1080/13683500.2015.1070801

10. Adenuga KI, Adenuga RO, Ziraba A, Mbuh PE. Healthcare augmentation: Social adoption of augmented reality glasses in medicine. ACM International Conference Proceeding Series 2019;71–74. doi: 10.1145/3328833.3328840

11. Han D-ID, Tom Dieck MC, Jung T. Augmented Reality Smart Glasses (ARSG) visitor adoption in cultural tourism. Leisure Studies Routledge; 2019 Sep 3;38(5):618–633. doi: 10.1080/02614367.2019.1604790

12. Prilla M, Blunk O, Osmers N, Janssen M. Social acceptance from the perspective of HMD users in small social settings – Observations from the field. CHI: Proceedings of the 1st Workshop on Challenges Using Head-Mounted Displays in Shared and Social Spaces 2019.

13. Zuidhof N, Ben Allouch S, Peters O, Verbeek PP. Anticipated Acceptance of Head Mounted Displays: A content analysis of YouTube comments. 2019 IEEE International Conference on Pervasive Computing and Communications Workshops, PerCom Workshops 2019 2019. p. 399–402. doi: 10.1109/PERCOMW.2019.8730658

14. Romina Sorko S, Komar J. Qualitative Acceptance Model of Augmented Reality from the Perspective of Personalists. Tehnički glasnik 2020;14(3):352–359. doi: 10.31803/tg-20200719183209

15. Holdack E, Lurie-Stoyanov K, Fromme HF. The role of perceived enjoyment and perceived informativeness in assessing the acceptance of AR wearables. Journal of Retailing and Consumer Services Elsevier Ltd; 2020;(November 2019):102259. doi: 10.1016/j.jretconser.2020.102259

16. Özdemir-Güngör D, Göken M, Basoglu N, Shaygan A, Dabić M, Daim TU. An Acceptance Model for the Adoption of Smart Glasses Technology by Healthcare Professionals. 2020. doi: 10.1007/978-3-030-27285-2_6ISBN:9783030272852

17. Larbaigt J, Lemercier C. An Evaluation of the Acceptability of Smart Eyewear for Plot Diagnosis Activity in Agriculture. Ergonomics in Design 2021;1–8. doi: 10.1177/10648046211018541

18. Martínez-Galdámez M, Fernández JG, Arteaga MS, Pérez-Sánchez L, Arenillas JF, Rodríguez-Arias C, Čulo B, Rotim A, Rotim K, Kalousek V. “Smart glasses evaluation during the COVID-19 pandemic: first-use on Neurointerventional procedures.” Clin Neurol Neurosurg Elsevier; 2021;106655. doi: 10.1016/j.clineuro.2021.106655

19. Zuidhof N, Ben Allouch S, Peters O, Verbeek P-P. Perspectives on the adoption and mediation implications of smart glasses: a qualitative focus group study in healthcare. submitted 2021;
