# Supplementary material for: Social Acceptance of Smart Glasses in Health Care: Model Evaluation Study of Anticipated Adoption and Social Interaction
Source: JMIR Form Res. 2025 Feb 11;9:e49610. doi: 10.2196/49610 (PMC11862762; doi:10.2196/49610)
Supplement: Multimedia Appendix 2 [file formative_v9i1e49610_app2.docx]

**Multimedia Appendix 2.** Item pool, descriptive statistics, factor loadings, squared multiple correlations, and Cronbach alpha of the observed indicators to explain and predict the anticipated use of smart glasses.

| Perspective from the framework (figure 2) | Aim | Source | *Construct* + adapted items | m (sd) | β | *R^2^* |
| --- | --- | --- | --- | --- | --- | --- |
| 1. Adoption (diffusion baseline) | Insight into the phase of diffusion of smart glasses | An individual’s decision is a process that occurs over time and consist of series of actions. Knowledge is the phase of exposing the individual to smart glasses and the gained understanding of how it functions.  Persuasion occurs actions are taken that lead to adopt or reject [31]. | *Knowledge* (α = .81)  KN1. The existence of smart glasses is known to me KN2. I know there are smart glasses  KN3. I understand what smart glasses are KN4. I can explain what smart glasses are  KN 5. I know what someone can do with smart glasses  KN 6. The functionalities of smart glasses are known to me  *Persuasion* (α = .91) PS1. I have actively searched for information about smart glasses  PS2. I looked up information about smart glasses PS3. I imagined how I would use smart glasses PS4. I have been thinking about how I would use smart glasses PS5. I discussed evaluations of smart glasses with peers  PS6. I have talked to others about smart glasses | m (sd) 3.95 (1.01)  4.03 (.96) 4.08 (.79) 3.97 (.88)  4.02 (.84) 3.86 (.97)  3.47 (1.27) 3.53 (1.26) 3.76 (1.09)  3.62 (1.15) 3.47 (1.13) 3.56 (1.29) | Β N/A | *R^2^*  N/A |
| 1. Adoption (professionals perspective)  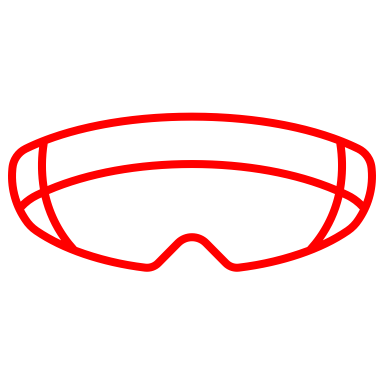  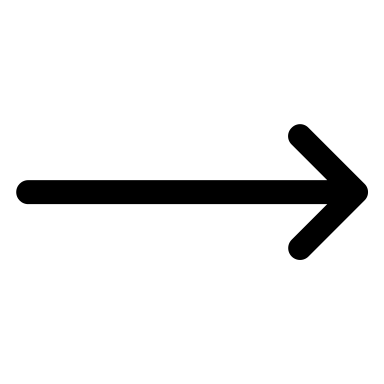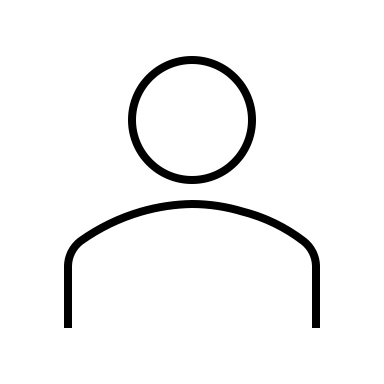 | Insight into the anticipated adoption of smart glasses | “the degree to which a person believes that using a particular system would enhance his or her job performance” [68] Usefulness [69]  “the degree to which a person believes that using a particular system would be free of effort” [68] Ease of use [69]    Person’s perception that most people who are important to him think he should or should not perform the behavior in question [70] [60] Social norm [71]  The degree to which use of an innovation is perceived to enhance one’s image or status in one’s social system.” [72]  “The degree to which a medium permits users to experience others as being psychologically present” (Lee et al., 2003) Impersonal, unsociable, cold, insensitive  [49]  The degree to which a person likes or dislikes the object [73] Attitude toward using AAL technology [71]  Behavioral intention to use the system (Ben Allouch, 2008; Jaschinski, 2018; Venkatesh & Bala, 2008b)  The use of smart glasses reduces social interaction due to less eye contact and social isolation of the user [28] Limited social interaction when using smart glasses [20] | *Perceived Usefulness* (α = .87)  PU1. Using smart glasses would improve my performance in my job  PU2. Using smart glasses in my job would increase my productivity  PU3. Using smart glasses would enhance my effectiveness in my job  PU4. I would find smart glasses useful in my job  PU5. Using smart glasses would be convenient in my work  *Perceived ease of use* (α =.77)  PEOU 1. I expect that interacting with smart glasses would be clear  PEOU 2. I expect that interacting with smart glasses does not require a lot of my mental effort  PEOU 3. I expect that I find smart glasses easy to use  PEOU 4. I expect to find it easy to get smart glasses to do what I want to do.  PEOU5. I think I will know quickly how to use smart glasses  *Subjective norm* (α =.76)  SN 1 People who influence my behaviour think that I should use smart glasses in the future.  SN2 People who are important to me think that I should use smart glasses in the future.  SN3 Most people who are important to me would have a positive opinion towards use of smart glasses  *Image:* (α =.85)  IMG1 People in my organization who use smart glasses have more prestige than those who do not.  IMG2 People in my organization who use smart glasses have a high profile.  IMG3 Having smart glasses is a status symbol in my organization.  *Lack of social presence* (α =.90)  LSP1.Using smart glasses during patient contact will make me impersonal  LSP2. Using smart glasses during patient contact will make me unsociable  LSP3. I will experience the contact with the patient as colder by using smart glasses  LSP4. Using smart glasses during patient contact will make me insensitive  *Attitude* (α =.81)  ATT1. Using smart glasses is a good idea  ATT2. Using smart glasses is valuable  ATT 3. I like the idea of using smart glasses  ATT4. Using smart glasses is/seems enjoyable  ATT5. Using smart glasses is wise  *Intention to use* (α =.82)  ITU1.In the future, I plan to use smart glasses  ITU2. In the future, I expect to use smart glasses  ITU3. I intend to use smart glasses in the future  *Social Isolation (from patient)* (α =.91)  SOI1.Using smart glasses reduces social interaction with my patient  SOI2. The use of smart glasses worsens social contact with the patient  SOI3. The use of smart glasses will reduce eye contact with the patient  SOI4. By using smart glasses I look less at my patient  SOI5. Wearing smart glasses makes me less approachable for the patient  SOI6. Using smart glasses results in more social distance with my patient | 3.85 (.98)  3.81 (.95)  3.88 (.96)  3.88 (1.01)  3.88 (.95)  3.98 (.85)  3.85 (.97)  3.98 (.85)  3.92 (.86)  3.98 (.84)  3.63 (.98)  3.70 (1.09)  3.89 (.90)  3.64 (1.05)  3.71 (1.08)  3.63 (1.10)  3.36 (1.21)  3.33 (1.26)  3.42 (1.21)  3.31 (1.26)  4.05 (.79)  4.08 (.88)  4.11 (.86) 4.03 (.86) 4.04 (.89)  3.88 (.96)  3.94 (.93) 3.93 (.96)  3.44 (1.10)  3.51 (1.25)  3.62 (1.12)  3.49 (1.13)  3.50 (1.14)  3.56 (1.15) | .78 .77 .72  .78  .71  .64  .60  .63  .71  .60  .80  .74  .63    .79  .80  .83 | .51  .61  .59  .52  .60  .50  .00 .41  .36  .40  .51  .36  .00  .63  .54  .40  .29 .63  .63  .70 |
|  |  |  |  |  | .82  .84  .78  .82    .75 .61  .70 .63 .67  .79 .76 .78  .84  .82  .69  .76  .81  .82 | .11  .67  .71  .60  .67  .89 .56  .37  .48  .40  .45  .96  .63  .58  .60  .05  .70  .67  .48  .58  .66  .68 |
|  |  | “Social presence in a mutual interaction with a perceived entity refers to the degree of initial awareness, allocated attention, the capacity for both content and affective comprehension, and the capacity for both affective and behavioral interdependence with said entity.”  [74] | *Decreased attentional allocation (towards patient)* (α =.92)  Imagine you are wearing smart glasses and have a face-to-face interaction with your patient:  DAA1. Smart glasses would make me easily distracted from my patient during a face-to-face interaction  DAA2. Smart glasses disrupt my attention to the patient  DAA3. I wouldn't be able to stay focused on my patient during our face-to-face interaction while wearing smart glasses DAA4. Because of smart glasses my attention is less focused on the patient  DAA5. My patient would not receive my full attention during the face-to-face interaction while wearing smart glasses  DAA6. By using smart glasses I have less attention for my patient | 3.46 (1.13)  3.42 (1.24)  3.36 (1.17)  3.44 (1.24)  3.45 (1.20)  3.32 (1.27) | .84  .84  .80  .80  .79  .85 | .00  .70  .70  .64  .64  .63  .72 |
| 2.Social interaction (professionals perspective)  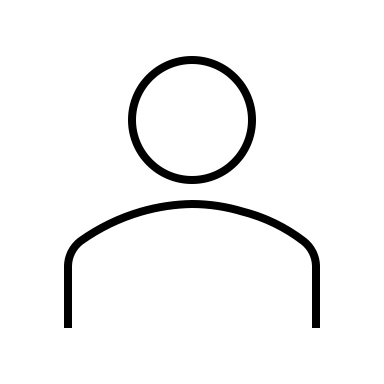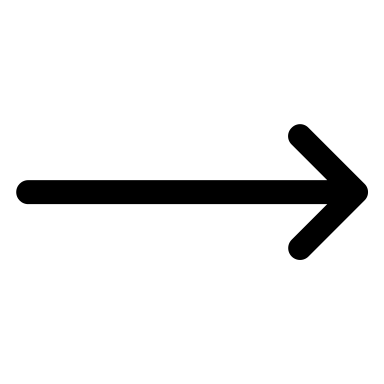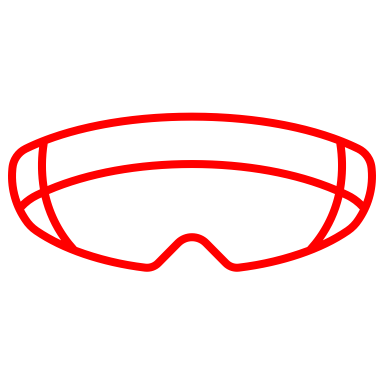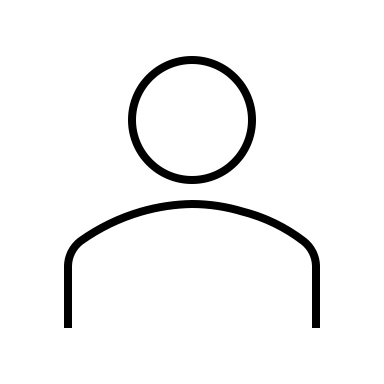 | 4. Insights in perceived influences from the perspective of a professional with smart glasses towards a patient without smart glasses | Influences in social interaction manifest by purpose of use, context of use the relationship with the user and emotions  (Zuidhof, 2021). For every task performed, they will be left with an impression: comfortable – uncomfortable, awkward – natural, relaxed or embarrassed, overall expression positive or negative impression of the task or technology  [42]  Tension, discomfort, arise when the purpose is unknown or behavior with smart glasses is misunderstood [28] | *Social acceptance: external location* (α =.75) Imagine you wear smart glasses and you provide care… - at a patients home (outpatient care) How would you feel towards your patient… SAEL1. Comfortable (5) SAEL2. Natural (5) SAEL3. Relaxed (5)  *Unfamiliar person* (α =.81) SAUP Imagine you wear smart glasses and you provide care… - to a patient you don’t know How would you feel towards your patient… SAUP1. Comfortable (5) SAUP2. Natural (5) SAUP3. Relaxed (5)  *Passive use* (α =.78) SAPU Imagine you wear smart glasses and you provide care… - **without** actively using the smart glasses How would you feel towards your patient… SAPU1. Comfortable (5) SAPU2. Natural (5) SAPU3. Relaxed (5) | 4.08 (.87) 3.88 (.93) 4.05 (.89)  3.94 (.94) 3.86 (.91) 3.88 (.93)  4.00 (.82) 4.02 (.93) 4.05 (.85) | .67  .77  .65  .73  .73  .71  .75  .66  .71 | .53  .45  .59  .43  .03  .54  .54  .50  .00  .56  .43  .50 |
| 3.Social interaction (imagined patients perspective)  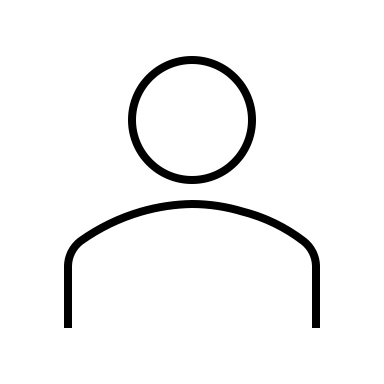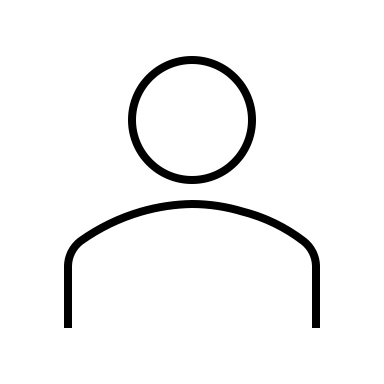  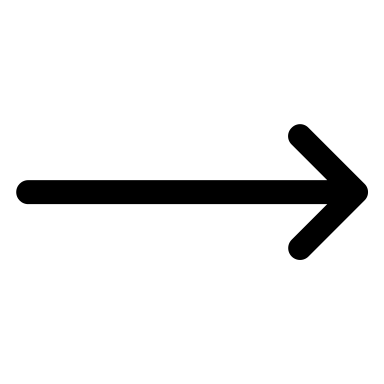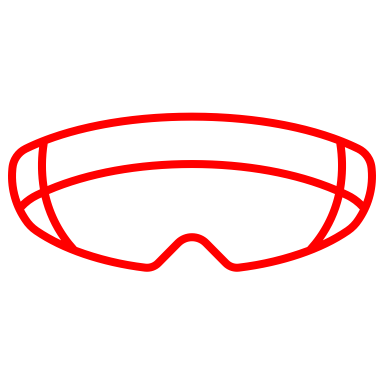  4.Sustained use intention (professionals perspective)  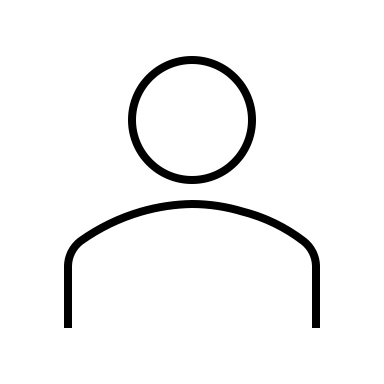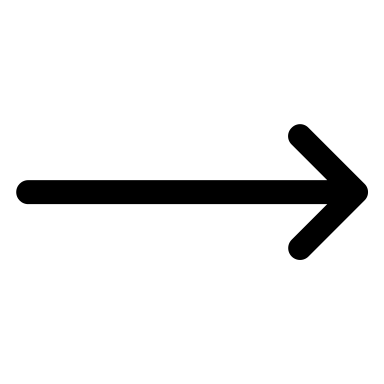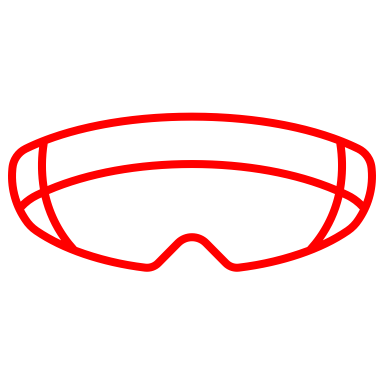 | 4.Insight in influences of social interaction by smart glasses usage on intention to use | Spectator’s social acceptance [42]. Consequences to the actor, consequences to other persons mediated through 1) the social structure, 2) the culture and 3) the civilization [52] | *Intention to use after imagined social nonacceptance by patients* (α =.92)  Indicate whether you would intend to use smart glasses in the following situations:  ISN1. I intend to use smart glasses in the future, even though my patient is not comfortable with smart glasses  ISN2. I intend to use smart glasses in the future, despite my patient feeling awkward with the use  ISN3. I intend to use smart glasses in the future, despite my patient being embarrassed about using smart glasses ISN4. I intend to use smart glasses in the future even though my patient is suspicious about smart glasses  ISN5. I intend to use smart glasses in the future despite my patient has an overall negative attitude toward smart glasses | 3.45 (1.21)  3.52 (1.12)  3.55 (1.14)  3.47 (1.13)  3.50 (1.21) | .81  .82  .84  .81  .86 | .65  .65  .67  .70  .66  .73 |
