# Supplementary material for: Social Acceptance of Smart Glasses in Health Care: Model Evaluation Study of Anticipated Adoption and Social Interaction
Source: JMIR Form Res. 2025 Feb 11;9:e49610. doi: 10.2196/49610 (PMC11862762; doi:10.2196/49610)
Supplement: Multimedia Appendix 3 [file formative_v9i1e49610_app3.docx]

**Multimedia Appendix 3.** Opening statement.

You are being invited to participate in a research study titled ‘The anticipated acceptance and social interaction of smart glasses’***.*** This study is being done by Niek Zuidhof, Oscar Peters, Peter-Paul Verbeek, and Somaya Ben Allouch from the Faculty of Behavioural, Management and Social Sciences at the University of Twente.

The purpose of this research study is to develop a research instrument to investigate both acceptance and social interaction and to get insight into how anticipated use is influenced by acceptance and social interaction. It will take you approximately 15 minutes to complete. The data will only be used in this study for statistical analyses and to make informed statements about the future use of smart glasses.

Your participation in this study is entirely voluntary and you can withdraw at any time. You are free to omit any questions, see contact details at the end of this page. For a fully completed questionnaire, you will receive $2.

We believe there are no known risks associated with this research study; however, as with any online-related activity, the risk of a breach is always possible. To the best of our ability, your answers in this study will remain confidential. We will minimize any risks by anonymizing the data (not traceable to a person) and storing the data in a central and secured server of the research institution. This research study has been submitted and approved by the ethics committee of the University of Twente.

A video is used once in the questionnaire, make sure you have your sound on. By clicking the button below you indicate that you have read the description of this study and agree to participate in this study.

We would like to thank you in advance for your participation!

Yours sincerely,

Niek Zuidhof

e.n.zuidhof@utwente.nl
